# Effects of stochastic resonance on reflexive and perceptual outcomes in chronic ankle instability

**DOI:** 10.3389/fspor.2025.1688251

**Published:** 2025-11-28

**Authors:** Annalee M. H. Friedman, Leif P. Madsen

**Affiliations:** 1 College of Health and Human Services, Department of Applied Medicine and Rehabilitation, Indiana State University, Terre Haute, IN, United States; 2 School of Public Health, Department of Kinesiology, Indiana University, Bloomington, IN, United States

**Keywords:** chronic ankle instability, lateral ankle sprain, cutaneous reflexes, stochastic resonance, sural nerve stimulation

## Abstract

**Introduction:**

Abnormal lower-limb cutaneous reflex characteristics following stimulation during gait have been observed among those with chronic ankle instability (CAI), which have been linked to perceptions of instability. These outcomes have the potential to serve as biomarkers of CAI or functional measures of sensorimotor recovery following lateral ankle sprain or clinical rehabilitation. However, no studies, to date, have identified a targeted intervention to regulate cutaneous reflexes in this population. Therefore, the purpose of this study was to explore the effects of stochastic resonance (SR) on lower-limb cutaneous reflexes and perceived instability during gait in those with CAI and healthy controls.

**Methods:**

Participants walked on a treadmill at 4 km/h while receiving random, non-noxious sural nerve stimulations throughout the stance phases of gait, reporting their perceived instability following each stimulation on an 11-point numeric rating scale. Mixed-factor ANOVAs were used to determine if a subthreshold stimulation applied to the ankle via an SR device affected ipsilateral lower leg reflexes and perceptions of ankle instability between groups.

**Results:**

Results indicate that the CAI group exhibited elevated PL facilitation and gastrocnemius reflex variability during midstance as well as increased perceptions of instability following sural stimulation.

**Discussion:**

SR may enhance consistency of cutaneous reflexes and reduce perceptions of instability following sural stimulation among those with CAI, indicating this intervention should be explored in a clinical setting as an accessible intervention to improve sensorimotor and patient-reported outcomes.

## 1 Introduction

Ankle ligament sprains are one of the most common injuries sustained by physically active people (1). Although often perceived as minor, insult to the lateral ankle ligaments can result in residual signs and symptoms that can ultimately diminish a person's health related quality of life and be a source of financial burden for the healthcare system by means of direct and indirect costs (2). Specifically, research has shown that upwards of 70% of people who sustain an acute ankle sprain can develop chronic ankle instability (CAI), a condition characterized by residual symptoms of perceived ankle instability and pain, impairments in proprioception and balance, an increased risk of recurrent sprains, and the development of early-onset osteoarthritis (3). Given the negative consequences of acute ankle sprains, strategies are needed to effectively reduce the public health burden of these chronic sequelae. One area of interest amongst researchers investigates how acute ankle sprains affect the neural control of human movement, and whether CAI may be associated with abnormal processing of afferent information relayed by mechanoreceptors in and around the ankle (4–6).

Cutaneous mechanoreceptors play a vital role in the maintenance of postural stability during both static and dynamic tasks (7–10). Information gathered from perturbation of peripheral nerves and afferents in the skin contribute to conceptualization of the physical environment in which humans navigate (8–11). When an unexpected cutaneous sensation occurs, afferent information is modulated at both the spinal and supraspinal levels based on characteristics of the sensation as well as the task and phase of movement during which the perturbation occurs (8–10, 12, 13). While cutaneous reflexes are generally consistent within younger populations, elderly adults have been found to exhibit depressed cutaneous reflex amplitudes which decreases postural stability and increases fall risk associated with aging (14). Given the postural and proprioceptive deficits frequently observed in people with CAI, researchers have more recently explored how cutaneous reflex alterations may be associated with the chronic symptoms that persist following lateral ankle sprains (6, 15–19).

Preliminary CAI studies investigated the excitability of middle latency cutaneous reflexes in the peroneus longus (PL) as people with and without a history of ankle sprains were in a seated position. The results of these studies found an exaggerated PL inhibition during sitting among those with CAI which was sustained over a 3-month period, indicating cutaneous reflexes may serve as a clinical biomarker for sensorimotor deficit commonly seen in this population (16, 17). Subsequent studies then incorporated more functional tasks and found that people with CAI experience altered cutaneous reflexes in the lateral gastrocnemius (LG) during the stance phase of gait. In healthy adults, inhibition of the LG middle latency reflex (80–120 ms) is considered normal following non-noxious sural nerve stimulation during stance (6, 18). This motor control mechanism allows weight to unload from the perturbed stance leg, transition to the contralateral limb, and helps maintain cyclical ambulation should postural control become compromised (20). However, those with CAI may not exhibit this protective unloading response as readily, with some studies reporting people with CAI lack this LG inhibition early in the stance phase, other studies found these LG reflex amplitudes, although present, were significantly more variable compared to healthy controls (6, 18, 19). Significant PL facilitation and subsequent eversion was observed in healthy controls during a drop-landing task which was absent among those with CAI (21). This suggests a lack of frontal plane stability of the ankle just prior to ground contact following a jump, which may leave this population at greater risk of recurrent LAS (21). Recent literature (19, 22–24) also indicates these reflex alterations may be linked to self-reported ankle function and perceptions of instability, hallmark symptoms of CAI which contribute to behavioral changes and reduced health-related quality of life (2, 15). Specifically, one study found greater variability in lower limb cutaneous reflexes predicted greater levels of perceived instability following sural nerve stimulation (19).

Finding interventions that effectively target these cutaneous reflex alterations post ankle sprain may be beneficial for clinicians looking to treat the chronic symptoms of CAI. Most recently, subthreshold mechanical vibration was identified as a potential intervention to improve cutaneous reflex modulation as it enhanced reflexes which contribute to postural control mechanisms while standing with a forward lean (25). In the context of cutaneous afferents, this vibration intervention elicits a phenomenon known as stochastic resonance (SR) whereby random, subthreshold (imperceptible) noise applied to the skin enhances the sensitivity of surrounding mechanoreceptors (i.e., Merkel cells, Ruffini endings, and Meissner and Pacinian corpuscles), which is thought to enrich sensory content from afferent stimuli processed in the spinal cord, subsequently improving motor output (26–29). Subthreshold vibration is thought to elevate resting membrane potential, thus increasing the number of activated cutaneous mechanoreceptors and subsequent information being transmitted to spinal interneurons (25). A 2019 study identified enhanced motoneuron pool excitability following a subthreshold vibration intervention applied to wrist flexor tendons, indicating this intervention may enhance functional outcomes via modulation at the spinal level (30). The authors highlight that neurophysiological responses to vibration presented in their study as well as others indicate this intervention may be sufficient to elicit the behavioral changes seen in various patient populations (30). Research over the last two decades has found SR improves a variety of functional outcomes related to sensorimotor function in a variety of patient populations (26, 31, 32) as well as postural stability in young healthy individuals, the elderly, and those with CAI (33–35). Considering the plasticity of cutaneous reflexes and the efficacy of stochastic resonance in improving reflexive and functional outcomes, exploring this intervention's effect on cutaneous reflex characteristics in a CAI population would shed light on its value in neuromuscular rehabilitation.

Therefore, the purpose of this study was to determine whether stochastic resonance affects cutaneous reflex amplitudes or variability of the peroneus longus and gastrocnemius muscles, as well as perceptions of ankle instability following cutaneous stimulation during gait among those with and without CAI. To further examine the clinical efficacy of SR for these outcomes, this study also sought to observe reflexive and perceptual characteristics post-SR intervention. We hypothesized that, compared to healthy controls, people with CAI will report higher levels of perceived ankle instability following non-noxious sural nerve stimulation during the stance phase of gait, which will be accompanied by highly variable reflex amplitudes in the PL and LG. Additionally, we hypothesized that the application of a commercially available SR device at the ankle will result in more consistent reflex amplitudes and reduced feelings of instability post stimulation in this patient population.

## 2 Methods

**Figure 1:**
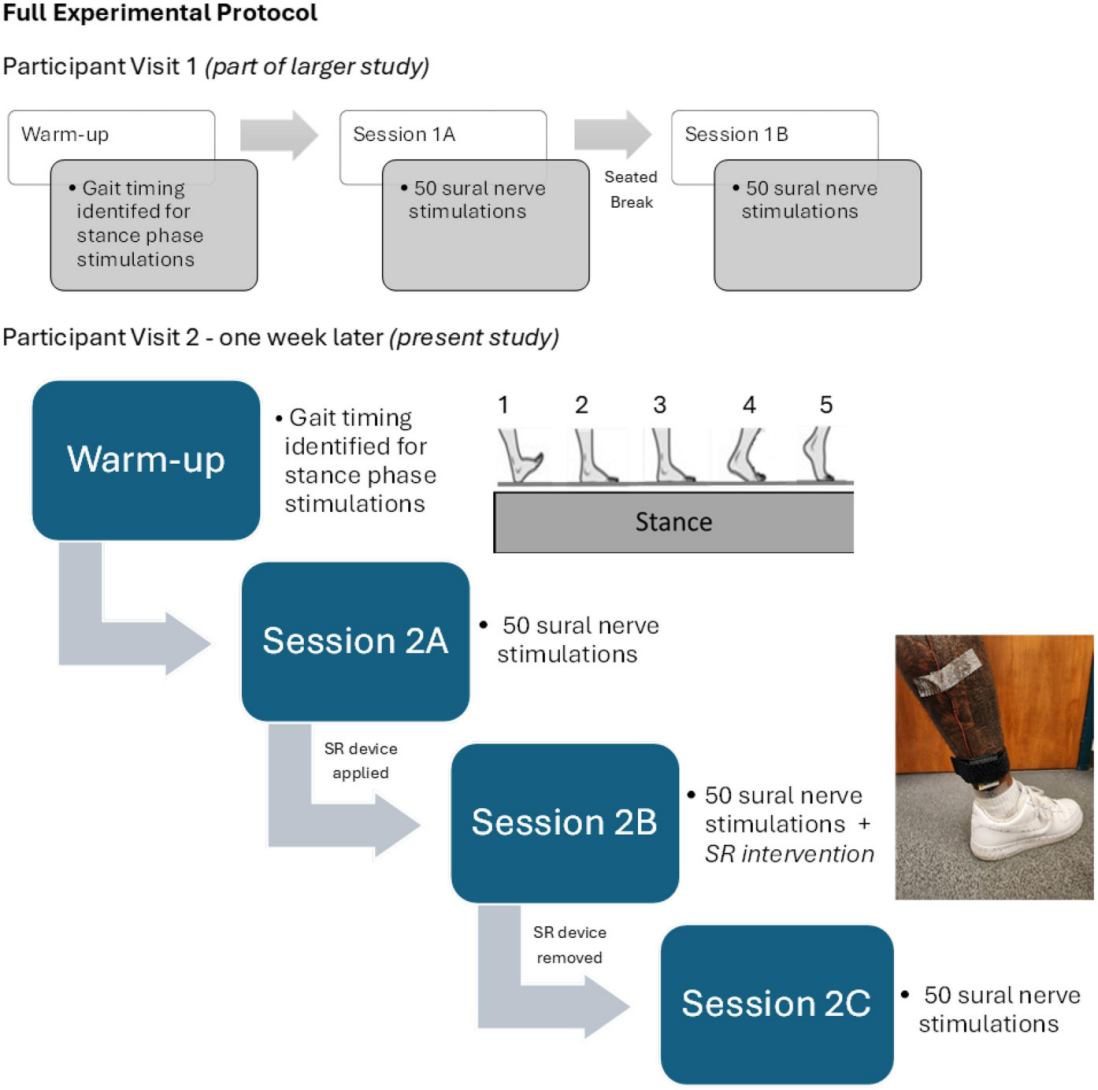
Experimental protocol for entire participant data collection. All participants attended two testing visits as a part of a larger study exploring reflexive and perceptual outcomes in individuals with CAI over a 1-week period. The gray flowchart illustrates the study protocol for the first visit (part of the larger study). (36, 37) The blue flowchart illustrates the second visit only, during which the SR intervention was investigated (the present study). Images depict the stance phases of the gait cycle during which simulations were administered and the positioning of the Accelera SR-100 device during Session 2B.

This study was part of a larger experiment, the methods of which have been previously published (see Figure 1) (36, 37). The following methods describe the experimental procedures relevant to this study's aims focusing on lower-limb cutaneous reflex characteristics and perceived instability during phases 1–5 of gait (stance) before, during, and after application of an SR intervention. This study was approved by the University's Institutional Review Board for the Protection of Human Subjects and was conducted in accordance with the Declaration of Helsinki.

### 2.1 Participants

All thirty-two individuals participating in the study were neurologically intact, physically active adults with no history of fractures or surgeries in the lower extremity, and no injuries within 6 weeks prior to data collection. All participants completed the Identification of Functional Ankle Instability (IdFAI) and Cumberland Ankle Instability Tool (CAIT) questionnaires to determine group assignment, a method endorsed by the International Ankle Consortium (37). Participants with a history of at least one acute LAS occurring at least 12 months prior to participation, an IdFAI score ≥11, and a CAIT score ≤24 in at least one limb were placed in the CAI group while participants with no history of LAS, scoring 0 on the IDFAI, and 30 on the CAIT were placed in the control group (see Table 1). The most affected limb (highest IDFAI & lowest CAIT score) was used as the test limb in CAI participants which was then matched in the control group based on limb dominance.

**Table 1:** Participant demographics and questionnaire scores (mean ± SD).

Characteristic	CAI Group (*n* = 16)	Control Group (*n* = 16)
Demographics		
Male	10	11
Female	6	5
Age	21.1 ± 5.03	21.2 ± 4.56
Height (cm)	171 ± 10.3	167 ± 8.02
Weight (kg)	70.7 ± 14.7	67.1 ± 13.2
Questionnaires		
Ankle Sprains	2.88 ± 1.99	0.00 ± 0.00
IdFAI Scores	20.6 ± 2.29	0.00 ± 0.00
CAIT Scores	17.2 ± 5.20	30.0 ± 0.00

### 2.2 Protocol

All participants attended two testing visits as a part of a larger study exploring reflexive and perceptual outcomes in individuals with CAI over a 1-week period. However, this study addresses data from the second visit only, during which the SR intervention was investigated (see Figure 1). During this visit, participants walked on a treadmill at 4 km/h. While walking for a 5-minute warm-up period, the average gait cycle timing was identified for each participant which was used to elicit stimulations across 5 equal time points (phases) of stance, phase 1 indicating heel strike through phase 5 indicating toe-off. Participants then continued walking while receiving random, non-noxious stimulations (2 times radiating threshold) to the sural nerve via a stimulating bar electrode (Ambu, Inc, Columbia, MD) affixed just posterior to the lateral malleolus. Following each stimulation, participants reported their feelings of instability on an 11-point numeric rating scale which was manually recorded for each trial. Ten stimulations were administered at each of the 5 phases of stance for a total of 50 stimulations per session. This round of stimulations constituted session 2A of the study—sessions 1A and 1B were part of the larger study exploring reflex measurement reliability and are discussed in another article (36).

After session 2A, participants took a seated break (approximately 5 min) and researchers secured an Accelera SR-100 wearable stochastic resonance device (Accelera, Inc., Biddeford, ME) to the participant's test limb. This commercially available device uses a piezoelectric crystal which vibrates randomly within a given range of frequencies attached to two transducers on either side of the device. The device was secured with a loop and fastener strap to the lateral aspect of the participant's ankle, proximal to the stimulating bar electrode and under the electrode leads to prevent lead tension or interference to the device's contact with the skin. Researchers turned on the device and modified vibration intensity using the Accelera IOS application (Accelera, Inc., Biddeford, ME). Starting at 0% the researchers slowly increased the vibration intensity until the participant reported they could feel vibration around the foot or ankle. To identify the precise perceptual threshold (PT), the intensity was then reduced until the participant could no longer perceive the vibration. The final device intensity was 90% of PT and remained on throughout the duration of the next walking task. Once the SR-100 was set-up, participants repeated the stimulated walking protocol and instability reports while walking at 4 km/h (session 2B).

Following session 2B, participants took another seated break, and the SR-100 was removed. The stimulated walking protocol and instability reports were then repeated once more without the device (session 2C). Muscle activity was measured continuously throughout all 3 sessions via bipolar surface electrodes (Delsys Inc, Natick, MA) affixed over the muscle bellies of the PL, LG and medial gastrocnemius (MG). All EMG, stimulation, and heel strike data were recorded using a Biopac MP160 acquisition system and Acqknowledge 5.0 software (Biopac Systems, Inc., Goleta, CA).

### 2.3 Data processing

Raw EMG data for the PL, MG, and LG were filtered at a 50 Hz low frequency cutoff and a 500 Hz high frequency cutoff and root mean square (RMS) was derived. Each session (2A, 2B, and 2C) was processed separately; ensemble averaged stimulated gait cycles represented 10 trials for each phase (1–5) within each session and ensemble averaged unstimulated gait cycles represented approximately 100 gait cycles for each session.

All waveforms were normalized as a percentage of maximum ensemble averaged unstimulated muscle activity. The unstimulated waveforms were then subtracted from stimulated waveforms to acquire a normalized net reflex waveform for each muscle at each phase. Middle-latency reflex (MLR) values (80–120 ms post-stimulation) were extracted from the net reflex waveform at each muscle for each of the 5 phases of stance, a time window which aligns with recent literature (38, 39). Average EMG muscle activity during this window was extracted from normalized stimulated and unstimulated waveforms for analysis of reflex variability and background muscle activity, respectively. All data processing was completed using AcqKnowledge 5.0 software (Biopac Systems, Inc., Goleta, CA).

### 2.4 Statistical analysis

Three-way mixed factor ANOVAs (one for the PL, MG, and LG) were used to analyze unstimulated muscle activity with 1 between-subjects factor at 2 levels (CAI and control groups), 1 within-subjects factor at 3 levels (session 2A, 2B, and 2C), and 1 within-subjects factors at 5 levels (phase of gait). Because background muscle activity can affect subsequent reflex amplitudes (40, 41), these analyses were completed to identify differences in background EMG between groups or sessions. When significant differences in background EMG mirrored significant differences in reflex amplitudes as revealed by reflex amplitude analyses (described later in this article), Pearson's correlations were run to determine whether background muscle activity affected subsequent motor output.

To identify differences in reflex amplitudes between sessions, 3 separate three-way mixed factor ANOVAs (one for each muscle) were used to analyze the normalized average reflex amplitudes with 1 between-subjects factor at 2 levels (CAI and control groups), 1 within-subjects factor at 3 levels (session 2A, 2B, and 2C), and 1 within-subjects factors at 5 levels (phase of gait). The same analyses were performed for the reflex variability data (standard deviation derived from the 10 trials of normalized stimulated data for each phase). Session, phase, or group main effects and interactions were used to determine if reflex amplitudes or variability were altered by the application of the SR-100 device.

Additionally, 5 two-way mixed factor ANOVAs (one for each phase of stance) were used to analyze the perceived instability reports with 1 between-subjects factor at 2 levels (CAI and control groups), and 1 within-subjects factor at 3 levels (session 2A, 2B, and 2C). Session or group main effects and interactions were used to determine if perceived instability was altered by the application of the Accelera SR-100 device in either group.

Normality of background, reflex, and variability data were assessed through inspection of group QQ plots for each muscle, at each phase. Greenhouse-Geisser corrections were used when the assumption of equal variances was not met throughout statistical analyses. Box and whisker plots for each variable were used to identify extreme outliers which were subsequently explored to determine whether the EMG values accurately represented muscle activity collected throughout the testing session. After screening outlier data points for potential errors during data collection, all analyses were run with and without remaining outliers to determine if removal affected statistical outcomes. All statistical analyses were conducted using IBM SPSS Version 29.0.1 (IBM Corporation, Armonk, NY) with significance set *a priori* at *p* ≤ .05.

## 3 Results

Electromyographical and patient-reported data from all 32 participants were analyzed for all three sessions of the study. Participant demographic information and questionnaire scores are reported in Table 1.

### 3.1 Background EMG

Removal of outliers (*n* = 2) identified for background EMG resulted in no change in statistical outcomes, therefore, all 32 participants were used in these analyses. A three-way ANOVA for the PL identified no significant three-way interaction (F_3.46,104_ = 1.64, *p* = .178). No session by group (F_2,60_=.672, *p* = .514) or phase by group (F_1.89,56.6_ = 3.04, *p* = .059) interactions were found, however, a significant phase by session (F_3.46,104_ = 5.17, *p* = .001) interaction was identified. Main effect for group was not significant (F_1,30_=.473, *p* = .497), but a significant session main effect was revealed (F_2,60_ = 3.913, *p* = .025), specifically, background PL activity was greater during session 2A [37.4%, 95%CI (35.0, 39.9)] and during session 2B [37.4%, 95%CI (34.9, 39.9)] compared to 2C [36.1%, 95%CI (33.8, 38.5)].

The three-way ANOVA for the MG revealed a significant three-way interaction (F_4.33,129_ = 2.97, *p* = .019) as well as significant phase by session (F_4.33,129_ = 4.812, *p* < .001) and session by group (F_2,60_ = 3.92, *p* = .025) interactions. No phase by group interaction was identified for the MG (F_1.57,47.1_ = 1.04, *p* = .346). A significant group main effect (F_1,30_ = 10.2, *p* = .003), specifically, background muscle activity in the MG was greater among the CAI group [32.0%, 95%CI (29.8, 34.3)] compared to controls [26.9%, 95%CI (24.7, 29.3)]. No session main effect was identified for this muscle (F_2,60_=.438, *p* = .628).

In the LG, three-way (F_3.40,102_ = 2.124, *p* = .094), phase by group (F_1.55,46.6_=.603, *p* = .511), and session by group (F_2,60_=.414, *p* = .663) interactions were not significant, however, a significant phase by session interaction (F_3.40,102_ = 6.82, *p* < .001) was identified. A significant session main effect was also identified (F_2,60_ = 3.63, *p* = .033), indicating background LG activity was greater during session 2A [32.1%, 95%CI (30.0, 34.2)] compared to session 2B [31.1%, 95%CI (29.2, 33.0)] and 2C [30.8%, 95%CI (29.0, 32.7)]. No group main effect was found for LG background analyses (F_1,30_=.353, *p* = .557).

Finally, three-way mixed factor ANOVAs revealed significant phase main effects (*p* < .001) for all muscles, indicating background EMG amplitudes varied throughout the gait cycle in accordance with the demands of this dynamic task, as anticipated.

### 3.2 Reflex amplitudes

Three-way mixed factor ANOVAs for reflex amplitudes revealed no significant three-way interactions for any muscle measured (PL: F_3.78,113_ = 1.56, *p* = .192, MG: F_3.83,115_ = 1.17, *p* = .328, LG: F_3.78,113_=.741, *p* = .559). Similarly, no two-way interactions (phase by group, session by group, or phase by session) were identified for any muscle analyzed.

**Figure 2:**
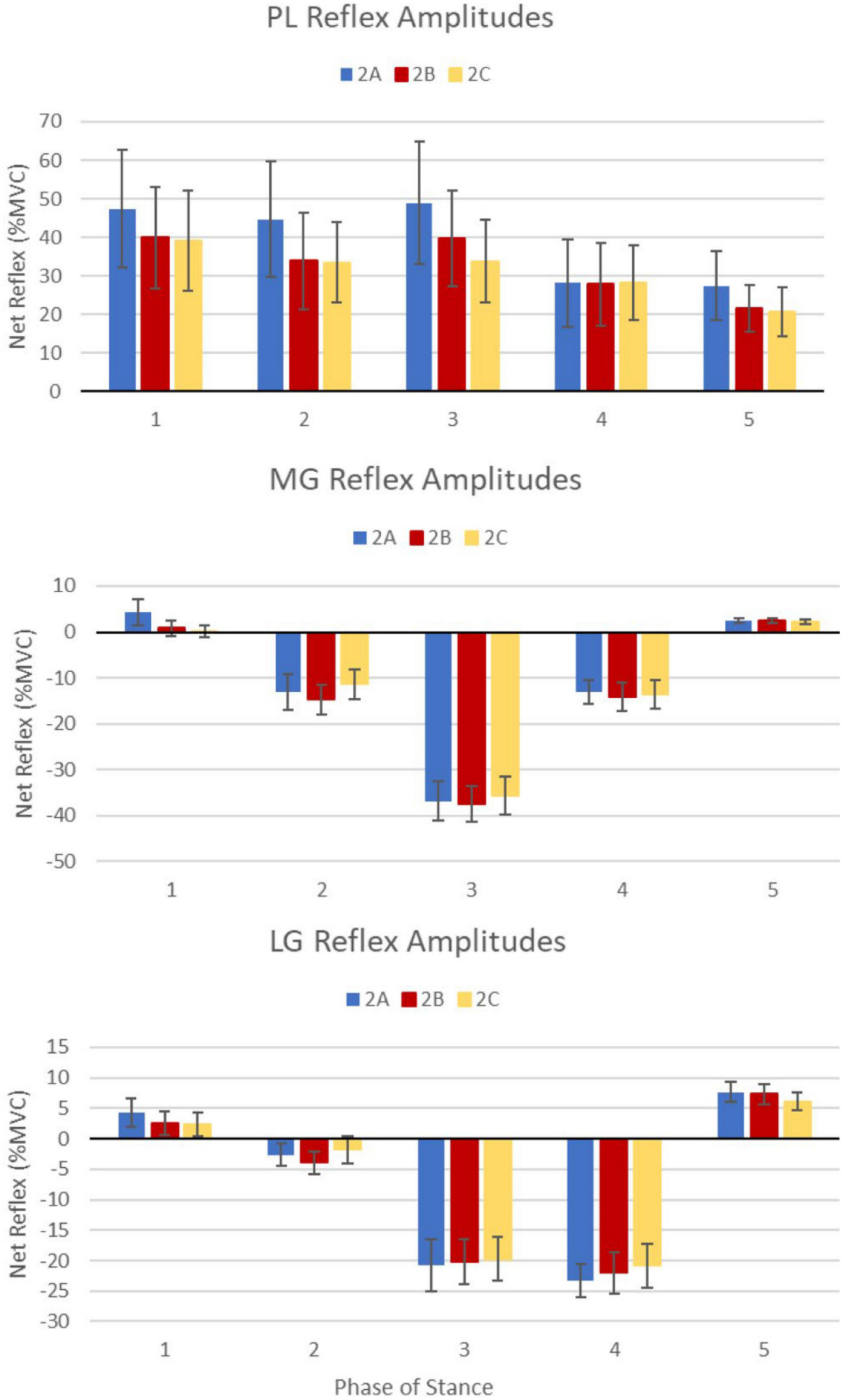
Cutaneous reflex patterns across stance for the PL, MG, and LG. Average net reflexes at each session for entire sample with standard error denoted. Phase main effects were identified for all muscles, indicating phase-dependent reflex amplitudes throughout stance.

Significant phase main effects for the PL (F_1.87,56.1_ = 3.99, *p* = .026), MG (F_2.02,60.6_ = 49.1, *p* < .001), and LG (F_2.26,67.8_ = 42.9, *p* < .001) indicating reflex amplitudes were phase-dependent throughout stance (see Figure 2). No group main effects were revealed for any muscle, but a session main effect was identified for the PL (F_1.27,38.1_ = 4.20, *p* = .038), specifically, greater reflex amplitudes were exhibited among all participants during session 2A [39.3%, 95%CI (13.1, 65.6)] compared to session 2B [32.6%, 95%CI (11.2, 54.1)] and session 2C [31.1%, 95%CI (11.3, 50.9)]. When running these analyses without reflex amplitude outliers (*n* = 6) removal of one control participants resulted in an insignificant session main effect for the PL. Since this participant was only an outlier for phase 5 of sessions 2A and 2C, and their net muscle activity was reflective of true reflexes, they were kept in the final analysis. Pearson's correlations revealed significant relationships between background PL activity and PL reflex amplitudes, however these were only correlated during phase 1 [r(30) = .374, *p* = .035] and phase 2 [r(30) = .427, *p* = .015] of session 2A.

### 3.3 Reflex variability

Three-way mixed factor ANOVAs for reflex variability revealed no significant three-way interactions for any muscle measured (PL: F_2.46,73.8_ = 1.50, *p* = .227, MG: F_3.14,94.4_ = .557, *p* = .653, LG: F_5.25,157_ = 1.66, *p* = .144). Session by phase interactions were identified for the MG (F_3.15,94.4_ = 3.01, *p* = .032) and LG (F_5.25,157_ = 3.11, *p* = .009), but not for the PL (F_2.46,73.9_ = 1.40, *p* = .253).

**Figure 3:**
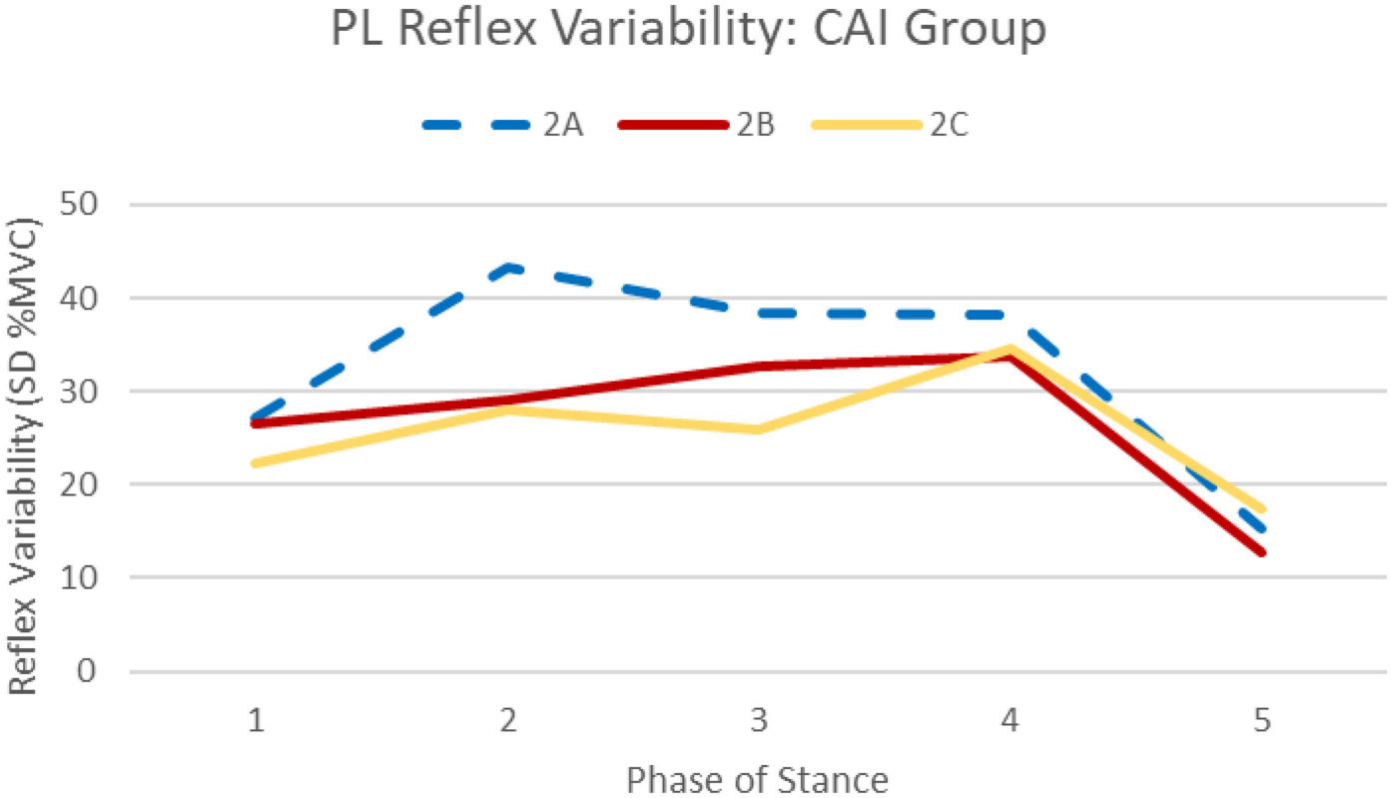
Average PL reflex variability (standard deviation of 10 trials at each phase) across stance at each session for the CAI group. Significant session by group interactions (*p* < .001) indicate greater PL reflex variability during session 2A (dashed) compared to sessions 2B and 2C (solid) among the CAI group.

**Figure 4:**
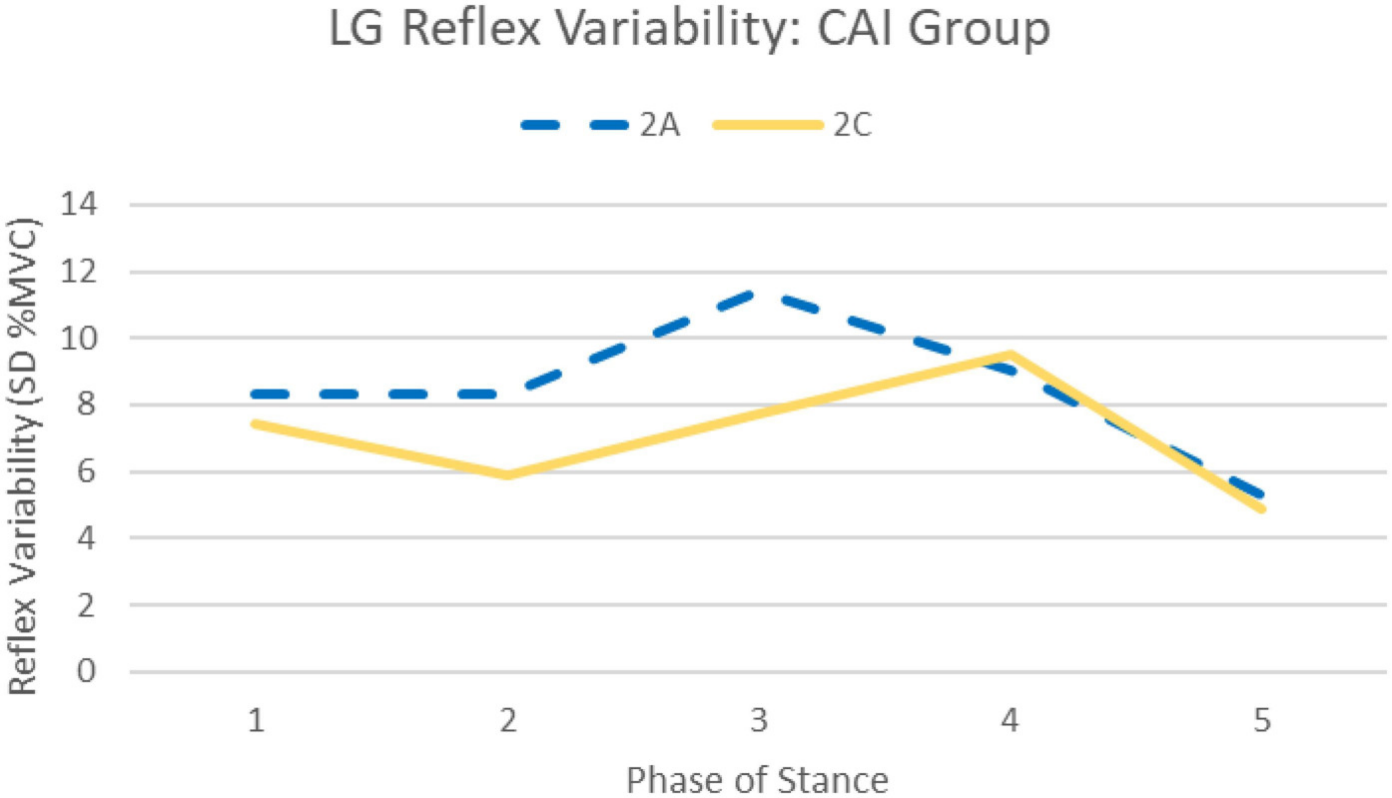
Average LG reflex variability (standard deviation of 10 trials at each phase) across stance at each session for the CAI group. Significant session by group interaction (*p* = 0.005) indicating greater reflex variability during session 2A (dashed) compared to 2C (solid) among the CAI group.

Session by group interactions were identified for the PL (F_1.17,35.3_ = 5.17, *p* = .024) and LG (F_2,60_ = 3.73, *p* = .030), specifically, total PL reflex variability was greater for the CAI group during session 2A [32.5%, 95%CI (20.4, 44.7)] compared to session 2B [26.9%, 95%CI (16.7, 37.2)] and 2C [25.7%, 95%CI (15.5, 35.9)] (see Figure 3). Total LG reflex variability was greater for the CAI group during session 2A (8.48%) compared to session 2C (7.09%, *p* = .005) (see Figure 4). No significant phase by group interactions were identified for any muscles analyzed.

**Figure 5:**
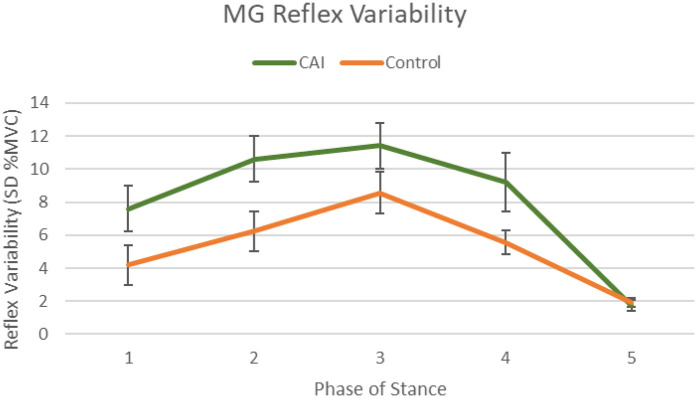
Combined MG reflex variability (standard deviation of 10 trials at each phase) across stance for all sessions with standard error denoted. Significant group main effect (*p* = 0.032) indicates the CAI group exhibited greater reflex variability throughout stance compared to controls, overall.

A significant phase main effects for all muscles (PL: F_2.15,64.2_ = 16.9, *p* < .001, MG: F_4,120_ = 24.9, *p* < .001 LG: F_2.65,79.5_ = 17.5, *p* < .001), indicating reflex variability also exhibited phase-dependency throughout stance. A group main effect was identified for the MG (F_1,30_ = 5.03, *p* = .032), indicating the CAI group exhibited greater variability throughout stance [8.12%, 95%CI (6.30, 9.93)] compared to controls [5.30%, 95%CI (3.49, 7.11)] (see Figure 5). No significant session main effects were found for any of the muscles evaluated. However, when these analyses were run with and without reflex variability outliers (*n* = 5) identified from normality analyses, removal of one control participant resulted in a significant session main effect for the PL (F_1.17,33.8_ = 5.076, *p* = .026) indicating PL reflex variability was greater during session 2A [24.9%, 95%CI(16.3, 33.4)] than sessions 2B [21.9%, 95%CI(15.1, 28.8)] and 2C [21.3%, 95%CI(15.1, 27.5)]. However, because this participant was only an outlier during phase 1 of session 2A and 2B and phases 1–4 of session 2C, and their net muscle activity reflected a true reflex, they were kept in the final analysis.

### 3.4 Perceived instability

Two-way mixed factor ANOVAs revealed group main effects for all phases of stance (*p* ≤ .002), indicating the CAI group reported greater levels of instability throughout stance. During phase 5, a session main effect was also identified (*p* = .001), specifically, average perceived instability for all participants was greater during session 2A (mean = 3.34) compared to session 2C (mean = 2.86, *p* = .024).

## 4 Discussion

### 4.1 Cutaneous reflex modulation

Phase-dependent reflex modulation was observed across the stance phase for all muscles measured during each of the 3 testing sessions in both groups (see Figure 2). PL facilitation is commonly seen during weight-bearing to prevent a potential inversion mechanism following lateral perturbation (6, 42, 43). Facilitation was exhibited by our sample most prominently during the first 3 phases of stance, from heel-strike into midstance, which was then reduced somewhat during late stance and the transition to swing. In the MG and LG, inhibition is expected during midstance, acting as a protective mechanism to prepare for an expedient transition to the contralateral limb if balance becomes compromised (6, 20, 44). This inhibition was exhibited most prominently in the MG at phase 3, and the LG at phases 3 and 4. During the transition to swing, gastrocnemius reflexes become facilitatory, acting to hasten swing and shift weight onto the unperturbed limb to improve postural stability and maintain a smooth movement cycle (6, 20, 44). Some facilitation was observed in the MG and, to a greater extent, in the LG during phase 5 of the gait cycle.

Optimally, reflex modulation following identical cutaneous stimulations should be consistent, allowing for an appropriate response to maintain postural control following every perturbation. A previous study exploring lower limb reflexes during gait attributed the lack of gastrocnemius inhibition seen among those with CAI to pronounced variability across sural stimulation trials (6). Our findings align with this supposition, as those with CAI exhibited greater MG variability across the stance phase compared to controls during all three testing sessions. Greater reflex variability in response to cutaneous perturbation may result in inadequate dynamic balance mechanisms which may contribute to perceived instability and greater injury risk often observed in this population (15, 19, 45).

### 4.2 Stochastic resonance intervention

**Figure 6:**
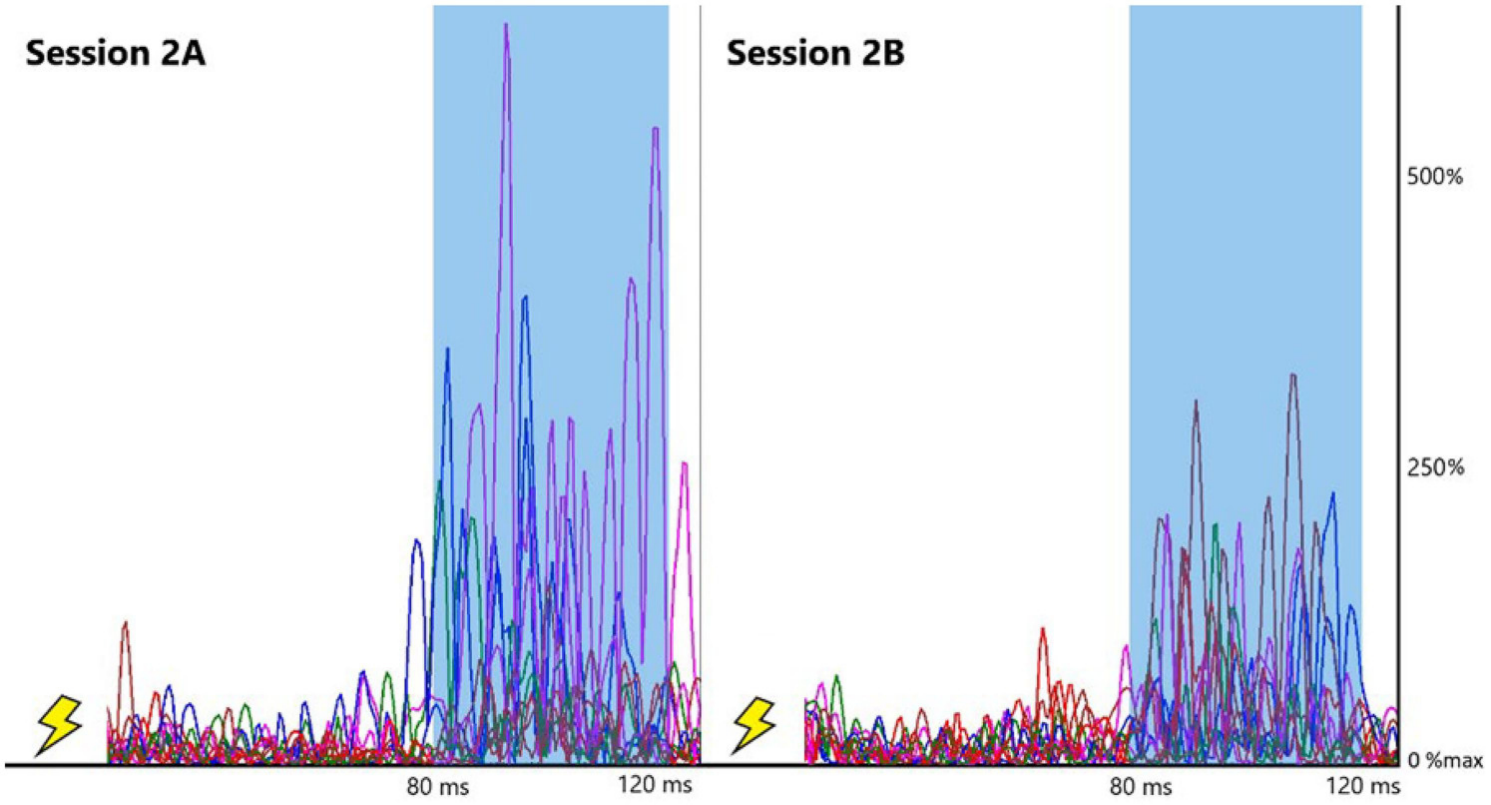
PL reflex variability reflex traces. Each line represents normalized EMG waveform for a stimulated trial (10 total) within one example CAI participant. Shaded areas represent middle-latency reflex window, 80–120 ms post-stimulation (artifact removed).

While phase-dependency was consistent across all testing sessions, reflex amplitudes were reduced in the PL, overall, from 2A to 2B (see individual example in Figure 6) and from 2B to 2C. It should be noted that background PL activity was also reduced across sessions during phases 1, 2, 3, and 5 which may have impacted the session main effects identified for the PL according to the principles of automatic gain compensation (40, 41). However, Pearson's correlations indicate background and reflex amplitudes are only correlated at phases 1 and 2 of session 2A. Previous work in our lab found excellent within-session reliability of PL reflex amplitudes (session 1A and 1B), indicating the reduction in PL reflex amplitudes observed during sessions 2B and 2C was likely a result of application of the SR intervention rather than attenuation across the testing period (36). Previous literature, as well as observations from our lab indicate some individuals with CAI exhibit more pronounced PL reflexive activity, suggesting a hyper-reactivity to cutaneous stimulation among this population (17, 37, 46). The reduction in PL facilitation observed in this study indicates the application of SR during gait may have corrected any hypersensitivity present among our mixed sample of healthy controls and CAI patients. However, considering no group by session interactions were observed, it is likely the intervention simply reduced PL facilitation across all participants, not just those with CAI. MG and LG reflex amplitudes did not change across testing sessions, indicating the SR intervention did not affect average gastrocnemius cutaneous reflexes across the stance phase of gait.

While the mechanisms underlying SR in neuronal systems are not fully understood, subthreshold stimulation to cutaneous afferents is thought to increase attunement of suprathreshold afferent information to the spinal cord, such that efferent responses are disseminated more quickly and accurately (28, 29). Several types of cutaneous mechanoreceptors likely contributed to the detection of afferent information, namely those responsible for perception of stretching of skin throughout gait (Ruffini and Merkel cells) and mechanical vibration from the SR device (Pacinian corpuscles), the latter of which are most sensitive to small displacements of the skin (11, 47). These cells communicate via electric signal which maintain a negative resting membrane potential (−70 mV), however, when applying mechanical or electrical noise just below this threshold, the barrier posed by the negative potential is overcome (29, 48). As a result, mechanoreceptors become sensitized to cutaneous stimuli, allowing for a more comprehensive representation of the physical environment and the limb within it.

This sensory “priming” [as described by Ross, et al. (35)] may explain the reduction in PL facilitation seen across sessions as the sural nerve stimulations were more accurately recognized as nonthreatening, resulting in lower levels of facilitation throughout stance. In addition to the reduction in PL reflexive amplitudes observed during stance, reduction in reflex variability was also noted across testing sessions in the CAI group only (an example of this reduction in an individual participant is illustrated in Figure 6). Our results also indicate PL reflex variability was reduced following removal of the SR device in the CAI group, suggesting these effects are maintained, at least for a short period, in this population (see Figure 3). This was also the case for LG reflex variability which was reduced from session 2A to 2C (see Figure 4).

A study exploring coordination training among CAI and control participants found SR applied over lower leg musculature and ankle ligaments in tandem with training improved time-to-stabilization measures during a jump landing task in the CAI group only, indicating effects of this intervention extended beyond the application period (49). While the mechanisms behind SR's effect on balance is not fully understood, it is thought that the enhanced sensitivity that SR provides affects CAI populations specifically as they often exhibit deficiencies in somatosensation (28, 50–54). We speculate that the reduction in reflex variability observed in the CAI group in our study may be similarly due to enhanced sensitivity or “priming”, providing for a more accurate motor response following each sural nerve stimulation throughout stance.

Interestingly, recent studies from our lab found reflex variability is linked to perceptions of instability (19, 37). Specifically, PL and LG variability can predict instability reports following sural stimulation during midstance (19). These results suggest variability in motor output following perturbation elicits feelings of instability during gait, which is reasonable considering the protective role reflex modulation plays during functional activity. This aligns with the findings of the present study, that is, significant reduction in perceived instability from session 2A to 2C, mirroring findings of PL and LG reflex variability reduction (see Figures 3, 4). As part of our larger study, the reliability of perceived instability reporting following cutaneous stimulation was evaluated to identify a threshold for minimal clinically important differences in this patient-reported outcome (36). Results of those analyses suggest ≥2.07 out of 10 indicates a beneficial reduction in perceived instability during perturbed stance (36). While a statistically significant reduction in instability was identified between sessions 2A and 2C, average group reporting was only reduced by 0.48 out of 10, indicating the observed change in this outcome may not be clinically meaningful. The statistically significant change in perceived instability found in this study may be due to rest time between testing sessions and not solely the SR intervention.

### 4.3 Clinical & research implications

While this study is the first to examine SR's effect on cutaneous reflexes during gait, this intervention has been shown to reduce variability in other functional outcome measures and populations. One study identified reduction in stride, stance, and swing time variability when elderly participants used vibrating insoles to elicit SR (55). Gait time variability was also greater among recurrent fallers compared to non-fallers in the elderly population, indicating this outcome may be a determinant of fall risk (55). Another study found application of SR attenuated physiologic tremor during an upper extremity motor task, reducing deviation of finger position within a visual target (56). Additionally, among those with CAI, a 2014 study found an SR intervention applied at 4 lower limb muscles regulated center of pressure velocity, an objective measure of postural control (57). Considering balance deficits are well-evidenced in this population, regulating these outcomes through SR may provide a direct mechanism for improvement of sensorimotor function (58–61).

The reflexes measured in previous studies in our lab (19, 36, 37) and the present study reflect autonomic modulation at the spinal level, however, conscious perception following these middle-latency reflexes also contributes by determining potential changes in voluntary motor control. A study exploring this relationship compared random sural nerve stimulations to self-induced stimulations during gait and found conscious anticipation of perturbation reduces facilitatory activity in lower limb muscles (62). In our study, the reduced PL facilitation seen following SR application and subsequent decreased instability reports may further illustrate the relationship between cortical and spinal modulation. Therefore, it is also important to evaluate and regulate perceptions of instability following reflexive modulation to maintain a smooth movement cycle and prevent inappropriate gait modifications. Unsurprisingly, those with CAI reported greater levels of instability following stimulation compared to controls across all sessions, hence, it may be useful to observe changes in this outcome measure in this population as a biomarker for neuromuscular recovery. Since perceived instability may also be affected by the SR intervention, it could be used to improve perceived instability either directly or indirectly through improvement of reflexive outcomes.

Considering the changes observed following application of an SR intervention, namely in those with CAI, future studies should continue to explore sensory interventions as treatment for chronic deficits often seen in this population. While perceptions of instability were reduced following the SR intervention, this was not considered a clinically meaningful change after a single session, therefore, future studies should continue to explore the effects of subthreshold vibration on this patient-reported outcome over longer durations or multiple testing sessions to determine whether clinically beneficial changes can be achieved. One such way to achieve this may be through a wearable SR device, similar to the one used in this study, utilized during physical activity to identify changes in patient-reported outcomes. SR interventions could also be employed throughout LAS rehabilitation to explore its use in improving functional outcomes related to appropriate and consistent reflex modulation such as balance and proprioception with the goal of preventing chronic deficits following initial injury, altogether.

### 4.4 Limitations

Several limitations of this study should be noted. First, while previous literature and findings from our own lab suggest cutaneous reflexes and perceived instability following stimulation can be reliably measured over various periods of time, it is possible that repeated sural nerve stimulation itself contributed to the observed changes in this study (47). Recent literature provides evidence for suprathreshold cutaneous stimulation as an intervention to improve spinal excitability and amplify cutaneous reflex amplitudes (63, 64). This may also explain why LG reflex variability was reduced from session 2A to 2C but not 2B as the quantity of sural nerve stimulations increased throughout testing. The subjective nature of perceived instability reporting may also pose a limitation to this study. To ensure accurate reporting, researchers employed an identical script to explain the 0–10 scale prior to each of the 3 testing sessions.

Second, this study did not employ a sham trial, so we may not be able to say for certain that the reflexive and perceptual changes seen during SR device application were not, at least in part, due to cutaneous sensation from the device and strap around the ankle. While previous literature (65, 66) presents mixed findings regarding the effects of ankle bracing on reflexive outcomes, we do not believe contact of the device with the skin alone was responsible for the results of this study. Specifically, because there were no instances of significant changes from session 2A to 2B only, indicating effects were sustained after removal of the device. While tactile effects were not likely confounders in this study, in future studies using the Accelera SR-100, a sham condition may be employed by turning the device intensity to 0 remotely rather than turning the device off (which requires a manual switch, so participants are unaware of which trial involves subthreshold vibration and which trial does not).

Third, it should be noted that the commercial device used for administering subthreshold vibration has not been directly tested to ensure the presence of the stochastic resonance phenomena in each of our participants, therefore, all EMG and perceptual findings are based on application of subthreshold vibration with random peak to peak frequency between 10 and 325 Hz, with a maximum displacement amplitude of ∼50 μm. The device was set to 90% of perceptual threshold as recommended in previous literature to target SR effects in clinical patients (26, 31, 55).

## 5 Conclusions

This study provides evidence that application of subthreshold vibration may be used among those with CAI to effectively normalize the cutaneous reflex characteristics, namely, reducing excessive PL facilitation and regulating PL and LG reflex amplitudes for better consistency across instances of perturbation. This intervention may also serve to reduce perceptions of instability, a hallmark symptom of CAI. By modifying reflexive activity to align with that of healthy controls, neuromuscular function may subsequently benefit, leading to better functional and patient-reported outcome measures.

## Data Availability

The raw data supporting the conclusions of this article will be made available by the authors, without undue reservation.

## References

[1] Lin C-I Houtenbos S Lu Y-H Mayer F Wippert P-M. The epidemiology of chronic ankle instability with perceived ankle instability-a systematic review. J Foot Ankle Res. (2021) 14(1):1–11. 10.1186/s13047-021-00480-w 34049565 PMC8161930

[2] Houston MN Hoch JM Hoch MC. Patient-reported outcome measures in individuals with chronic ankle instability: a systematic review. J Athl Train. (2015) 50(10):1019–33. 10.4085/1062-6050-50.9.01 26332028 PMC4641540

[3] Herzog MM Kerr ZY Marshall SW Wikstrom EA. Epidemiology of ankle sprains and chronic ankle instability. J Athl Train. (2019) 54(6):603–10. 10.4085/1062-6050-447-17 31135209 PMC6602402

[4] Duysens J Levin O. Ankle sprains: getting off on the wrong foot. Exerc Sport Sci Rev. (2010) 38(3):143–9. 10.1097/JES.0b013e3181e4ebdd 20577063

[5] Duysens J Hoogkamer W Levin O. Is there “arthrogenic inhibition” of cutaneous reflexes in subjects with functional ankle instability? Clin Neurophysiol. (2013) 124(7):1264–6. 10.1016/j.clinph.2013.02.018 23567073

[6] Madsen LP Kitano K Koceja DM Zehr EP Docherty CL. Effects of chronic ankle instability on cutaneous reflex modulation during walking. Exp Brain Res. (2019) 237(8):1959–71. 10.1007/s00221-019-05565-4 31129695

[7] Duysens J Tax A Trippel M Dietz V. Increased amplitude of cutaneous reflexes during human running as compared to standing. Brain Res. (1993) 613(2):230–8. 10.1016/0006-8993(93)90903-Z 8186969

[8] Zehr EP Stein RB. What functions do reflexes serve during human locomotion? Prog Neurobiol. (1999) 58(2):185–205. 10.1016/S0301-0082(98)00081-1 10338359

[9] Panek I Bui T Wright A Brownstone R. Cutaneous afferent regulation of motor function. Acta Neurobiol Exp (Wars). (2014) 74(2):158–71. 10.55782/ane-2014-1982 24993626

[10] McGlone F Reilly D. The cutaneous sensory system. Neurosci Biobehav Rev. (2010) 34(2):148–59. 10.1016/j.neubiorev.2009.08.004 19712693

[11] Abraira VE Ginty DD. The sensory neurons of touch. Neuron. (2013) 79(4):618–39. 10.1016/j.neuron.2013.07.051 23972592 PMC3811145

[12] Stein RB. Reflex modulation during locomotion: functional significance. Adv Psychol. (1991) 78:21–36. 10.1016/S0166-4115(08)60736-0

[13] Jenner J Stephens J. Cutaneous reflex responses and their central nervous pathways studied in man. J Physiol (Lond). (1982) 333(1):405–19. 10.1113/jphysiol.1982.sp014461 7182471 PMC1197256

[14] Peters RM McKeown MD Carpenter MG Inglis JT. Losing touch: age-related changes in plantar skin sensitivity, lower limb cutaneous reflex strength, and postural stability in older adults. J Neurophysiol. (2016) 116(4):1848–58. 10.1152/jn.00339.2016 27489366 PMC5144713

[15] Hertel J Corbett RO. An updated model of chronic ankle instability. J Athl Train. (2019) 54(6):572–88. 10.4085/1062-6050-344-18 31162943 PMC6602403

[16] Futatsubashi G Sasada S Ohtsuka H Suzuki S Komiyama T. History-dependent changes in the recovery process of the middle latency cutaneous reflex gain after ankle sprain injury. Eur J Appl Physiol. (2016) 116(3):459–70. 10.1007/s00421-015-3292-8 26560108

[17] Futatsubashi G Sasada S Tazoe T Komiyama T. Gain modulation of the middle latency cutaneous reflex in patients with chronic joint instability after ankle sprain. Clin Neurophysiol. (2013) 124(7):1406–13. 10.1016/j.clinph.2013.01.029 23541471

[18] Madsen LP Friedman AM Docherty CL Kitano K Koceja DM. Middle and long latency cutaneous reflexes during the stance phase of gait in individuals with and without chronic ankle instability. Brain Sci. (2024) 14(12):1225. 10.3390/brainsci14121225 39766424 PMC11727029

[19] Friedman AMH Madsen LP. Perceived ankle instability and cutaneous reflex modulation during gait. Physiol Rep. (2023) 11(22):e15880. 10.14814/phy2.15880 37994398 PMC10665776

[20] Zehr E Stein R Komiyama T. Function of sural nerve reflexes during human walking. J Physiol (Lond). (1998) 507(1):305–14. 10.1111/j.1469-7793.1998.305bu.x 9490858 PMC2230764

[21] Heimark NE Friedman AM Kitano K Madsen LP. The role of sural nerve reflexes during drop-landing in subjects with and without chronic ankle instability. Exp Brain Res. (2023) 241(6):1–15. 10.1007/s00221-023-06636-3 37204505

[22] Thompson CS Hiller CE Schabrun SM. Altered spinal-level sensorimotor control related to pain and perceived instability in people with chronic ankle instability. J Sci Med Sport. (2019) 22(4):425–9. 10.1016/j.jsams.2018.10.009 30442546

[23] Harkey M McLeod MM Terada M Gribble PA Pietrosimone BG. Quadratic association between corticomotor and spinal-reflexive excitability and self-reported disability in participants with chronic ankle instability. J Sport Rehabil. (2016) 25(2):137–45. 10.1123/jsr.2014-0282 25759960

[24] Kim K-M Hart JM Saliba SA Hertel J. Relationships between self-reported ankle function and modulation of Hoffmann reflex in patients with chronic ankle instability. Phys Ther Sport. (2016) 17:63–8. 10.1016/j.ptsp.2015.05.003 26541975

[25] Sharma T Peters RM Bent LR. Subthreshold electrical noise applied to the plantar foot enhances lower-limb cutaneous reflex generation. Front Hum Neurosci. (2020) 14:351. 10.3389/fnhum.2020.00351 33005140 PMC7479210

[26] Lynn J Wolf A Bridges T Pottanat Z Spivey S Rolin O. Effects of stochastic resonance stimulation on manual function in children with hemiplegic cerebral palsy: a pilot clinical trial. PM R. (2022) 15(3):302–13. 35187840 10.1002/pmrj.12788

[27] Martınez L Pérez T Mirasso CR Manjarrez E. Stochastic resonance in the motor system: effects of noise on the monosynaptic reflex pathway of the cat spinal cord. J Neurophysiol. (2007) 97:4007–16. 10.1152/jn.01164.2006 17428901

[28] Woo MT Davids K Liukkonen J Orth D Chow JY Jaakkola T. Effects of different lower-limb sensory stimulation strategies on postural regulation—a systematic review and meta-analysis. PLoS One. (2017) 12(3):e0174522. 10.1371/journal.pone.0174522 28355265 PMC5371369

[29] Gammaitoni L Hänggi P Jung P Marchesoni F. Stochastic resonance. Rev Mod Phys. (1998) 70(1):223. 10.1103/RevModPhys.70.223

[30] Tenan MS Tweedell AJ Haynes CA Passaro AD. The effect of imperceptible Gaussian tendon vibration on the hoffmann reflex. Neurosci Lett. (2019) 706:123–7. 10.1016/j.neulet.2019.05.018 31085290

[31] Khaodhiar L Niemi JB Earnest R Lima C Harry JD Veves A. Enhancing sensation in diabetic neuropathic foot with mechanical noise. Diabetes Care. (2003) 26(12):3280–3. 10.2337/diacare.26.12.3280 14633814

[32] Kaut O Allert N Coch C Paus S Grzeska A Minnerop M Stochastic resonance therapy in Parkinson’s disease. NeuroRehabilitation. (2011) 28(4):353–8. 10.3233/NRE-2011-0663 21725168

[33] Ross SE Arnold BL. Postural stability benefits from training with stochastic resonance stimulation in stable and unstable ankles. Athl Train Sports Health Care. (2012) 4(5):207–12. 10.3928/01484834-20120731-03

[34] Priplata A Niemi J Salen M Harry J Lipsitz LA Collins J. Noise-enhanced human balance control. Phys Rev Lett. (2002) 89(23):238101. 10.1103/PhysRevLett.89.238101 12485044

[35] Ross S Arnold B. Noise-enhanced dynamic single leg balance in subjects with functional ankle instability. J Sport Health Sci. (2012) 1(2):102–6. 10.1016/j.jshs.2012.06.001

[36] Friedman AM Madsen LP. Reliability of reflex measurements and perceived instability following cutaneous stimulation during gait. J Electromyogr Kinesiol. (2024) 80:102958. 10.1016/j.jelekin.2024.102958 39657426

[37] Friedman AMH Madsen LP. Reflexive and perceptual characteristics as functional outcome measures of chronic ankle instability. Foot Ankle Int. (2025) 46(7):774–83. 10.1177/10711007251338279 40432161

[38] Madsen LP Kitano K Koceja DM Zehr EP Docherty CL. Modulation of cutaneous reflexes during sidestepping in adult humans. Exp Brain Res. (2020) 238(10):2229–43. 10.1007/s00221-020-05877-w 32710371

[39] Futatsubashi G Sasada S Ohtsuka H Suzuki S Komiyama T. Misencoding of ankle joint angle control system via cutaneous afferents reflex pathway in chronic ankle instability. Exp Brain Res. (2022) 240(9):1–11. 10.1007/s00221-022-06406-7 35764722

[40] Matthews P. Observations on the automatic compensation of reflex gain on varying the pre-existing level of motor discharge in man. J Physiol (Lond). (1986) 374(1):73–90. 10.1113/jphysiol.1986.sp016066 3746703 PMC1182707

[41] Pruszynski JA Kurtzer I Lillicrap TP Scott SH. Temporal evolution of “automatic gain-scaling”. J Neurophysiol. (2009) 102(2):992–1003. 10.1152/jn.00085.2009 19439680 PMC2724331

[42] Konradsen L Voigt M Hojsgaard C. Ankle inversion injuries: the role of the dynamic defense mechanism. Am J Sports Med. (1997) 25(1):54–8. 10.1177/036354659702500110 9006692

[43] Gutierrez GM Kaminski TW Douex AT. Neuromuscular control and ankle instability. PM R. (2009) 1(4):359–65. 10.1016/j.pmrj.2009.01.013 19627919

[44] Lamont EV Zehr EP. Task-specific modulation of cutaneous reflexes expressed at functionally relevant gait cycle phases during level and incline walking and stair climbing. Exp Brain Res. (2006) 173(1):185–92. 10.1007/s00221-006-0586-4 16821052

[45] Delahunt E Remus A. Risk factors for lateral ankle sprains and chronic ankle instability. J Athl Train. (2019) 54(6):611–6. 10.4085/1062-6050-44-18 31161942 PMC6602396

[46] Santos MJ Liu H Liu W. Unloading reactions in functional ankle instability. Gait Posture. (2008) 27(4):589–94. 10.1016/j.gaitpost.2007.08.001 17889541

[47] Hao J Bonnet C Amsalem M Ruel J Delmas P. Transduction and encoding sensory information by skin mechanoreceptors. Pflügers Arch. (2015) 467:109–19. 10.1007/s00424-014-1651-7 25416542

[48] Wellens T Shatokhin V Buchleitner A. Stochastic resonance. Rep Prog Phys. (2003) 67(1):45. 10.1088/0034-4885/67/1/R02

[49] Ross SE Guskiewicz KM. Effect of coordination training with and without stochastic resonance stimulation on dynamic postural stability of subjects with functional ankle instability and subjects with stable ankles. Clin J Sport Med. (2006) 16(4):323–8. 10.1097/00042752-200607000-00007 16858216

[50] Konradsen L. Factors contributing to chronic ankle instability: kinesthesia and joint position sense. J Athl Train. (2002) 37(4):381.PMID: 12937559 PMC164369

[51] Nakasa T Fukuhara K Adachi N Ochi M. The deficit of joint position sense in the chronic unstable ankle as measured by inversion angle replication error. Arch Orthop Trauma Surg. (2008) 128(5):445–9. 10.1007/s00402-007-0432-6 17874250

[52] Refshauge KM Kilbreath SL Raymond J. Deficits in detection of inversion and eversion movements among subjects with recurrent ankle sprains. J Orthop Sports Phys Ther. (2003) 33(4):166–76. 10.2519/jospt.2003.33.4.166 12723673

[53] Xa X Chen Z Xuan W Tao W Jin Z Hua Y. Force sense deficits in chronic ankle instability: a systematic review and meta-analysis. PM R. (2022) 15(6):780–9. 10.1002/pmrj.12833 35532066

[54] Hoch MC McKeon PO Andreatta RD. Plantar vibrotactile detection deficits in adults with chronic ankle instability. Med Sci Sports Exerc. (2012) 44(4):666–72. 10.1249/MSS.0b013e3182390212 21959910

[55] Galica AM Kang HG Priplata AA Andrea D Starobinets SE Sorond OV Subsensory vibrations to the feet reduce gait variability in elderly fallers. Gait Posture. (2009) 30(3):383–7. 10.1016/j.gaitpost.2009.07.005 19632845 PMC2745077

[56] Trenado C Amtage F Huethe F Schulte-Mönting J Mendez-Balbuena I Baker SN Suppression of enhanced physiological tremor via stochastic noise: initial observations. PLoS One. (2014) 9(11):e112782. 10.1371/journal.pone.0112782 25397577 PMC4232445

[57] Glass SM Ross SE Arnold BL Rhea CK. Center of pressure regularity with and without stochastic resonance stimulation in stable and unstable ankles. Athl Train Sports Health Care. (2014) 6(4):170–8.

[58] Docherty CL McLeod TCV Shultz SJ. Postural control deficits in participants with functional ankle instability as measured by the balance error scoring system. Clin J Sport Med. (2006) 16(3):203–8. 10.1097/00042752-200605000-00003 16778539

[59] Hertel J Olmsted-Kramer LC. Deficits in time-to-boundary measures of postural control with chronic ankle instability. Gait Posture. (2007) 25(1):33–9. 10.1016/j.gaitpost.2005.12.009 16446093

[60] McKeon PO Hertel J. Spatiotemporal postural control deficits are present in those with chronic ankle instability. BMC Musculoskelet Disord. (2008) 9(1):76. 10.1186/1471-2474-9-76 18518994 PMC2438356

[61] Hertel J Braham RA Hale SA Olmsted-Kramer LC. Simplifying the star excursion balance test: analyses of subjects with and without chronic ankle instability. J Orthop Sports Phys Ther. (2006) 36(3):131–7. 10.2519/jospt.2006.36.3.131 16596889

[62] Baken B Nieuwenhuijzen P Bastiaanse C Dietz V Duysens J. Cutaneous reflexes evoked during human walking are reduced when self-induced. J Physiol (Lond). (2006) 570(1):113–24. 10.1113/jphysiol.2005.095240 16269436 PMC1464299

[63] Pearcey GE Zehr EP. Repeated and patterned stimulation of cutaneous reflex pathways amplifies spinal cord excitability. J Neurophysiol. (2020) 124(2):342–51. 10.1152/jn.00072.2020 32579412 PMC7500381

[64] Sun Y Zehr EP. Sensory enhancement amplifies interlimb cutaneous reflexes in wrist extensor muscles. J Neurophysiol. (2019) 122(5):2085–94. 10.1152/jn.00324.2019 31509473 PMC6879954

[65] Sefton J Hicks-Little C Koceja D Cordova M. Effect of inversion and ankle bracing on peroneus longus Hoffmann reflex. Scand J Med Sci Sports. (2007) 17(5):539–46. 10.1111/j.1600-0838.2006.00593.x 17076833

[66] Nishikawa T Grabiner MD. Peroneal motoneuron excitability increases immediately following application of a semirigid ankle brace. J Orthop Sports Phys Ther. (1999) 29(3):168–76. 10.2519/jospt.1999.29.3.168 10322590

